# Impact on smoking of England's 2012 partial tobacco point of sale display ban: a repeated cross-sectional national study

**DOI:** 10.1136/tobaccocontrol-2015-052724

**Published:** 2016-02-22

**Authors:** Mirte A G Kuipers, Emma Beard, Sara C Hitchman, Jamie Brown, Karien Stronks, Anton E Kunst, Ann McNeill, Robert West

**Affiliations:** 1 Department of Public Health, Academic Medical Center, University of Amsterdam, Amsterdam, The Netherlands; 2 Department of Addictions, Institute of Psychiatry, Psychology and Neuroscience, King's College London, London, UK; 3 Department of Clinical, Educational and Health Psychology, University College London, London, UK; 4 Department of Epidemiology and Public Health, Health Behaviour Research Centre, University College London, London, UK

**Keywords:** Advertising and Promotion, Public policy, Socioeconomic status

## Abstract

**Background:**

A partial tobacco point of sale (PoS) display ban was introduced in large shops (>280 m^2^ floor area) in England on 6 April 2012. The aim of this study was to assess the medium-term effects of the partial tobacco PoS display ban on smoking in England.

**Methods:**

Data were used from 129 957 respondents participating in monthly, cross-sectional household surveys of representative samples of the English adult population aged 18+ years from January 2009 to February 2015. Interrupted-time series regression models assessed step changes in the level of current smoking and cigarette consumption in smokers and changes in the trends postban compared with preban. Models were adjusted for sociodemographic variables and e-cigarette use, seasonality and autocorrelation. Potential confounding by cigarette price was accounted for by time, as price was almost perfectly correlated with time.

**Results:**

Following the display ban, there was no immediate step level change in smoking (−3.69% change, 95% CI −7.94 to 0.75, p=0.102) or in cigarette consumption (β −0.183, 95% CI −0.602 to 0.236). There was a significantly steeper decline in smoking post display ban (−0.46% change, 95% CI −0.72 to −0.20, p=0.001). This effect was demonstrated by respondents in manual occupations (−0.62% change, 95% CI −0.72 to −0.20, p=0.001), but not for those in non-manual occupations (−0.42, 95% CI −0.90 to 0.06, p=0.084). Cigarette consumption declined preban period (β −0.486, 95% CI −0.633 to −0.339, p<0.001), but no significant change in cigarette consumption trend was observed (β 0.019, 95% CI −0.006 to 0.042, p=0.131).

**Conclusions:**

The partial tobacco PoS display ban introduced in England in April 2012 did not lead to an immediate decline in smoking, but was followed by a decline in the trend of smoking prevalence that could not be accounted for by seasonal factors, e-cigarette use or price changes.

## Introduction

Tobacco point of sale (PoS) display bans are relatively new policy measures that prohibit shops from displaying tobacco products. PoS display bans intend to strengthen existing policies prohibiting tobacco advertising. Iceland, Thailand, Canada, Australia, Ireland, Norway, Finland, New Zealand and the UK are among those countries that have bans on PoS display of tobacco products. The removal of tobacco displays could affect smoking by reducing smoking cues for smokers and recent ex-smokers, and by reducing the advertisement function of tobacco pack designs. A reduction in smoking cues might prevent impulse purchase^1–3^ and might, therefore, prevent relapse in recent ex-smokers and reduce cigarette consumption in smokers.^1^ ^4–7^ A reduction in tobacco advertisement through packs might diminish the image of tobacco being an available, normal, and recognisable product^8^ ^9^ and might, therefore, prevent smoking uptake in young people.^10–12^

There is limited evidence on the effect of PoS bans on smoking behaviour. Data from Ireland suggested that there were no significant short-term changes in prevalence among adults^13^ and that tobacco sales did not decrease noticeably in the first year after the ban was introduced.^14^ However, among young people in Australia, smoking, cigarette brand awareness, recall of PoS displays, and the overestimation of peer smoking significantly declined 2 years after the introduction of the ban.^15^ Positive effects from the denormalisation of tobacco use were also found in Norway.^16^ Australian smokers showed a reduction in impulse purchase of cigarettes after the display ban was introduced.^17^ ^18^ Impulse purchase also decreased in Canada.^18^ To date, there have not been any comprehensive longer term evaluation studies of PoS display bans that measured the impact on smoking prevalence or other smoking outcomes in the general population.

Evaluation studies of national tobacco control policies increasingly emphasise the effects of tobacco control policies on socioeconomic inequalities in smoking behaviour.^19^ ^20^ Since individuals from a lower social grade are more likely to initiate smoking and less likely to successfully quit,^21^ ^22^ it is important to identify policies that are at least equally effective across social groups (‘equity neutral’), or policies that are more effective in lower social groups (‘equity positive’). To date, tobacco taxation has been identified as having an equity positive impact on smoking.^20^ However, as an increase in tobacco price might magnify income inequalities and might promote poverty due to high spending on tobacco,^23^ ^24^ identifying additional policies that affect smoking in low-*socioeconomic* status groups is important.

The aim of this study was to assess whether the introduction of the partial PoS display ban in April 2012 in England resulted in a reduction in smoking prevalence and cigarette consumption in smokers, using data from the Smoking Toolkit Study (STS). A secondary aim was to assess the equity impact of the partial PoS display ban by comparing the effects between individuals in manual and non-manual occupations. The English partial PoS tobacco display ban came into force on 6 April 2012, banning all tobacco displays from larger shops (shops with a relevant floor area exceeding 280 m^2^).^25^ ^26^

## Methods

### Data and study population

Data were collected as part of the ongoing STS, a national survey of tobacco use in the general population of England. Each month, a new sample of approximately 1800 adults aged ≥16 years is selected using a form of random location sampling. Individuals complete a face-to-face computer-assisted household interview survey with a trained interviewer. The STS samples have been shown to be nationally representative in their sociodemographic composition and proportion of smokers in the population of 16+ years. Full details of the STS methods have been described elsewhere.^27^ Ethical approval was granted by the University College London ethics committee.

In the current study we used the data from January 2009 to February 2015. Any early effects of the second phase of the PoS display ban (in small shops) introduced in April 2015 therefore did not interfere with the results. Preban and postban periods were approximately equal in length, and met requirements of segmented regression analysis of at least 24 monthly data points before and after the intervention, with at least 100 cases for each data point.^28^

We included 129 957 individuals out of a total of 133 268. We excluded respondents with missing smoking status (N=126), and those under 18 years of age (N=3185) because they could not legally buy tobacco. For the analysis of cigarette consumption, we included 28 053 cigarette smokers, excluding 663 smokers who smoked products other than manufactured or hand-rolled cigarettes and 320 smokers with missing information on cigarette consumption.

### Measurements

#### Smoking outcome variables

Current smoking was measured with the question ‘Do you smoke or have you ever smoked?’ Respondents answering, ‘I smoke cigarettes (including hand-rolled) every day’, ‘I smoke cigarettes (including hand-rolled), but not every day’, or ‘I do not smoke cigarettes at all, but I do smoke tobacco of some other kind’ were considered current smokers. Respondents answering ‘I have stopped smoking completely in the last year’, ‘I have stopped smoking completely more than a year ago’, or ‘I have never been a smoker’ were non-smokers.

Cigarette consumption measured among current cigarette or hand-rolled smokers only was defined as the number of cigarettes smoked per day. Respondents were asked to estimate how many cigarettes they smoked per day, per week or per month. If consumption was reported per week or month, values were divided by 7 or 30, respectively. Respondents with values of over 40 cigarettes per day were considered outliers and were excluded (N=152). Cigarette consumption was treated as a continuous variable in all analyses and expressed as cigarettes per day.

#### Time variables

We distinguished the periods as pre and postintroduction of the partial PoS display ban that are coded as, respectively, 0 and 1. Preban period was defined as January 2009 to March 2012, and postban period was defined as April 2012 to February 2015. Time was measured as the months throughout the study period and was divided by 12 to represent the change in outcomes over a year rather than a month. The slope was defined as 0 before the introduction of the ban and each month after the introduction, by increments of 1 up to 34 (1, 2, 3 etc). To control for seasonality (month-of-year effects), the month within the year (‘calendar month’) was coded, with January coded as 1 to December coded 12.

#### Potential confounders

##### Sociodemographic variables

Age (in years), gender (males vs females) and social grade were assessed. Social grade was defined according to the National Readership Survey system of demographic classification. The five categories included ‘professional/managerial (AB)’, ‘clerical (C1)’, ‘skilled manual (C2)’, ‘semi-skilled manual (D)’, and ‘very low paid or unemployed (E)’. Non-manual occupation (AB and C1) was distinguished from manual occupation (C2, D and E).

##### Electronic cigarette prevalence

A significant potential confounder was the rapid increase in use of electronic cigarettes (e-cigarettes) from 2012 onwards.^29^ E-cigarettes are associated with increased smoking cessation rates^30^ and reduced cigarette consumption.^31^ E-cigarette use prevalence was measured monthly in the STS from April 2011 onwards, among smokers and recent (past year) ex-smokers, with the question ‘Are you using any of the following?’ Response options included ‘electronic cigarettes’. Prevalence of e-cigarette use was negligible prior to 2011 and was assumed to be 0 for January 2009 to March 2011. Six STS waves in 2012 and 2013 did not include the question on the use of e-cigarettes, and the average prevalence of the neighbouring waves was imputed.

##### Price of cigarettes

The average real price paid for a pack containing 20 manufactured cigarettes was derived from January 2009 to February 2015 Nielsen data on tobacco sales. Nielsen Scantrack digitally registers all tobacco sales from a selection of smaller and larger shops throughout Great Britain.^32^ From this sample, Nielsen estimated the total sales for Great Britain. Separate sales data for England were not available for the entire study period. We included sales information on all brands that were defined as single packs of 20 manufactured cigarettes. All packs with over 20 or under 20 cigarettes were excluded, as well as multipack cartons and roll-your-own tobacco. The average real price paid for a pack containing 20 cigarettes was determined by dividing the total value of tobacco sales (in £) by the total number of packs sold in each month.

### Statistical analysis

Data were analysed in R V.3.2.0. All data and analyses were unweighted and exact prevalence statistics will, therefore, differ marginally from previous STS publications (for more details, see Fidler *et al*^27^). Descriptive statistics are given for the overall sample and for the periods before and after the introduction of the PoS display ban.

An interrupted-time series design was used to assess the effect of the PoS ban on current smoking and cigarette consumption. Data were analysed with segmented regression using generalised additive models (GAM; R package mgvc),^33^ as applied by previous studies with a similar design.^34–36^ A quasi-binomial family was specified because there was overdispersion for current smoking.^37^ A log link function, rather than the traditional logit link function for logistic regression, was applied so that relative risks could be reported. For the GAMs with cigarette consumption as the outcome variable, the Gaussian family with identity link was used. Each GAM modelled the trend in the dependent variable in the preintervention period, any immediate step change in the dependent variable when the PoS ban occurred, and any change in the trends in the postban period relative to the preban period.

GAM models were adjusted in a stepwise manner. Model 0 was unadjusted; model 1 was adjusted for sociodemographic characteristics (age, gender and social grade); model 2 was additionally adjusted for e-cigarette use; and in model 3, a smooth term was added to control for any regular seasonal pattern in the outcomes of interest. For the smooth term, a maximum of 12 points was used (one for each month) and cyclic cubic regression splines specified. Parsimonious models provided the same pattern of results as the fully controlled models. Price of cigarettes was not included because of collinearity (R=0.99) with time, and the potential confounding effect of price was, therefore, largely accounted for by time.

Autocorrelation was assessed with the Durbin-Watson statistic autocorrelation function and partial autocorrelation function. There was a moderate amount of autoregressive 1 autocorrelation. To allow models for smoking status to run with adjustments for autocorrelation, the smoking status variable was coded as a count variable reflecting the number of smokers per 1000 individuals per month, and analysed using segmented Poisson regression. Autocorrelation was accounted for by including random components at the level of months within calendar years, and considered individual survey waves as the lower level by using generalised additive mixed models (GAMM; R package mgvc).^33^ There was no evidence for high overdispersion. Previous concerns have been raised regarding the use of GAMMs in R with binary data when the binomial family is specified, given that penalised quasi-likelihood is used and often fails when autocorrelation is specified in the mgvc package.^38^ Poisson regression has been employed previously to assess the impact of national tobacco control policies on smoking behaviour;^34^ this allows for autocorrelation to be adjusted for without the limitations introduced by specifying the binomial family. Adjustment for autocorrelation with cigarette consumption as the outcome was analysed at the individual level, again specifying the Gaussian distribution and identifying the link function.

The fully adjusted models was stratified according to social grade. Interactions were tested between social grade and, respectively, the preban trend, the step level change and the change in trend to test whether the impact of the ban differed by social grade. Interaction analyses were not adjusted for autocorrelation due to the complexity of the models.

## Results

Table 1 shows the description of the study population. Age and gender showed similar distributions before and after the introduction of the PoS ban. Social grade showed a shift towards higher social grade postban period, with a lower proportion of very low paid and unemployed, and a higher proportion of clerical class individuals. In the total study period, 22.1% were smokers (95% CI 21.8 to 22.3), and smoking was more prevalent in the preban period (23.2, 95% CI 22.9 to 23.5) than the postban period (20.7, 95% CI 20.4 to 21.1). Cigarette smokers smoked an average of 12.3 cigarettes per day (95% CI 12.2 to 12.4), and there was a decrease in consumption between the two periods.

**Table 1:** Description of the study population in the total study period, and before and after the introduction of the partial PoS display ban in England (unweighted data)

	Prevalence (%) with 95% CI		
	Total study period (January 2009—February 2015)	Prepartial PoS ban (January 2009—March 2012)	Postpartial PoS ban (April 2012—February 2015)
In the total population, N	131 552	71 405	60 147
Age (years)			
18–24	12.6, 12.4 to 12.8	11.3, 11.1 to 11.6	14.1, 13.8 to 14.3
25–34	16.4, 16.2 to 16.6	16.5, 16.2 to 16.8	16.2, 15.9 to 16.5
35–44	16.5, 16.3 to 16.7	17.5, 17.2 to 17.7	15.4, 15.1 to 15.7
45–54	15.6, 15.4 to 15.8	15.9, 15.6 to 16.2	15.2, 14.9 to 15.5
55–64	14.5, 14.3 to 14.7	14.4, 14.2 to 14.7	14.5, 14.3 to 14.8
65+	24.5, 24.3 to 24.7	24.4, 24.1 to 24.7	24.6, 24.2 to 24.9
Sex			
Female	51.9, 51.6 to 52.2	53.3, 52.9 to 53.6	50.2, 49.8 to 50.6
Male	48.1, 47.8 to 48.4	46.7, 46.4 to 47.1	49.8, 49.4 to 50.2
Social grade			
Professional/managerial (AB)	20.0, 19.8 to 20.2	19.8, 19.5 to 20.1	20.2, 19.9 to 20.5
Clerical (C1)	28.0, 27.8 to 28.2	26.8, 26.5 to 27.1	29.4, 29.1 to 29.8
Skilled manual (C2)	20.5, 20.3 to 20.7	19.8, 19.5 to 20.1	21.2, 20.9 to 21.6
Semiskilled manual (D)	15.2, 15.0 to 15.4	15.0, 14.7 to 15.3	15.4, 15.1 to 15.7
Very low paid or unemployed (E)	16.4, 16.2 to 16.6	18.6, 18.3 to 18.9	13.7, 13.4 to 14.0
E-cigarette prevalence*	1.84, 1.83 to 1.86	0.23, 0.22 to 0.23	3.77, 3.76 to 3.78
Current smoking			
Never-smoker or ex-smoker	77.9, 77.7 to 78.2	76.8, 76.5 to 77.1	79.3, 78.9 to 79.6
Current smoker	22.1, 21.8 to 22.3	23.2, 22.9 to 23.5	20.7, 20.4 to 21.1
In current smokers, N	28 053	16 005	12 048
Cigarette consumption (cigarettes per day)*	12.3, 12.2 to 12.4	12.8, 12.7–12.9	11.6, 11.4 to 11.7

*Mean, 95% CI.

PoS, point of sale.

**Figure 1:**
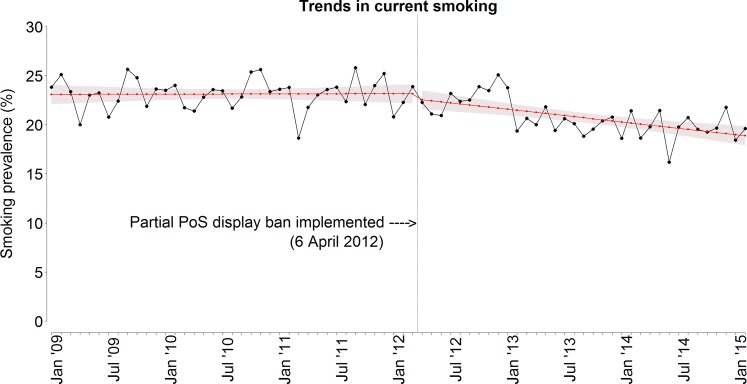
Crude trend and unadjusted fitted trend of current smoking (in %). The vertical line indicates the timing of the introduction of the partial PoS tobacco display ban in England on 6 April 2012. PoS, point of sale.

**Figure 2:**
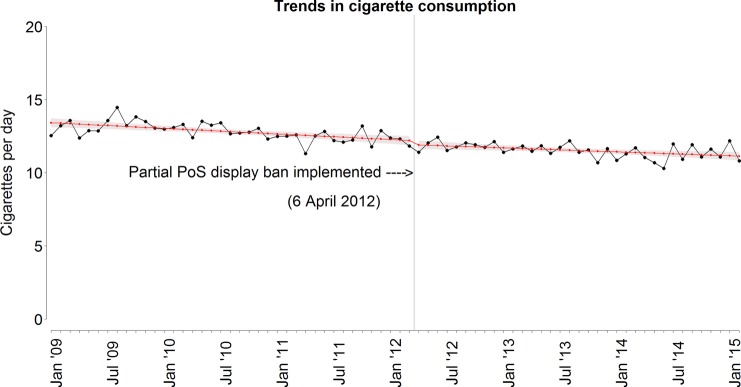
Crude trend and unadjusted fitted trend of cigarette consumption (in cigarettes per day). The vertical line indicates the timing of the introduction of the partial PoS tobacco display ban in England on 6 April 2012. PoS, point of sale.

Figure 1 presents the trends in current smoking. Current smoking decreased by approximately five percentage points over the total period, and this trend appears to be declining more steeply in the second period. Figure 2 presents the trends in cigarette consumption. The number of cigarettes smoked per day shows a linear decrease with a total decrease of about two cigarettes per day over the study period.

Table 2 presents the results of the segmented regression analysis for current smoking. The unadjusted model (model 0) shows that there was no decline in smoking before the introduction of the PoS ban (0.15% change, 95% CI −1.04 to 1.63, p=0.846). In the unadjusted model, there was no immediate step level change in smoking after the ban was introduced (−2.30% change, 95% CI −6.19 to 1.76, p=0.263). There was a significantly steeper declining trend in smoking after versus before the ban of PoS display (−0.53% change, 95% CI −0.73 to −0.33, p<0.001). This steeper decline in smoking remained significant after controlling for seasonality (−0.46% change, 95% CI −0.72 to −0.20, p=0.001), and was confirmed in the Poisson model that controlled for autocorrelation (−0.56% change, 95% CI −0.82 to −0.29, p<0.001).

**Table 2:** Results of the segmented regression analysis for current smoking presenting the trend in smoking previous to the introduction of the PoS display ban, the immediate step level change in smoking prevalence, and the change in the smoking trend after the PoS ban was introduced compared with the preban period trend

	Trend pre-PoS display ban			Step level change			Change in trend		
	Percentage change	95% CI	p Value	Percentage change	95% CI	p Value	Percentage change	95% CI	p Value
Log binomial models									
Model 0: unadjusted	0.15	−1.04 to 1.63	0.846	−2.30	−6.19 to 1.76	0.263	−**0.53**	−**0.73 to** −**0.33**	**<0.001**
Model 1: +sociodemographics*	−0.93	−2.31 to 0.47	0.190	−2.25	−5.96 to 1.61	0.249	−**0.21**	−**0.40 to** −**0.02**	**0.029**
Model 2: +e-cigarettes	−**1.68**	−**3.19 to** −**0.14**	**0.032**	−**4.54**	−**8.66 to** −**0.22**	**0.039**	−**0.41**	−**0.67 to** −**0.15**	**0.002**
Model 3: +seasonality	−**2.07**	−**3.59 to** −**0.52**	**0.009**	−3.69	−7.94 to 0.75	0.102	−**0.46**	−**0.72 to** −**0.20**	**0.001**
Poisson models									
Model 0: unadjusted	0.14	−2.04 to 2.37	0.902	−1.94	−7.77 to 4.26	0.531	−**0.53**	−**0.82 to** −**0.24**	**<0.001**
Model 1: adjusted for seasonality	−0.21	−2.34 to 1.96	0.849	−0.49	−6.35 to 5.73	0.874	−**0.56**	−**0.84 to** −**0.27**	**<0.001**
Model 2: +autocorrelation	−0.23	−2.21 to 1.80	0.826	−0.46	−5.95 to 5.34	0.873	−**0.56**	−**0.82 to** −**0.29**	**<0.001**

Significant associations are indicated in **bold** (p<0.05).

*Age, gender and social grade.

PoS, point of sale.

Table 3 presents the results of the segmented regression analysis for cigarette consumption. In all models, we found a significantly declining trend in cigarette consumption before the introduction of the PoS display ban (fully adjusted model: β −0.486, 95% CI −0.633 to −0.339, p<0.001). The step level change (β −0.183, 95% CI −0.602 to 0.236, p=0.392) and the change in trend (β 0.019, 95% CI −0.006 to 0.042) were not statistically significant: there was a consistent declining trend in cigarette consumption over the entire period under study (as was found in figure 2).

**Table 3:** Results of the segmented regression analysis for cigarette consumption presenting the trend in cigarette consumption previous to the introduction of the PoS display ban, the immediate step level change in cigarette consumption, and the change in the trend after the PoS display ban was introduced compared with the preban period trend

	Trend pre-PoS ban			Step level change			Change in trend		
	β	95% CI	p Value	β	95% CI	p Value	β	95% CI	p Value
Model 0: unadjusted	**−0.388**	**−0.520 to −0.255**	**<0.001**	**−**0.264	**−**0.627 to 0.101	0.156	0.010	**−**0.008 to 0027	0.282
Model 1: +sociodemographics*	**−0.464**	**−0.593 to −0.335**	**<0.001**	**−**0.148	**−**0.502 to 0.205	0.411	**0.022**	**0.005 to 0.039**	**0.011**
Model 2: +e-cigarettes	**−0.473**	**−0.615 to −0.330**	**<0.001**	0.176	**−**0.580 to 0.227	0.392	0.020	**−**0.003 to 0.043	0.091
Model 3: +seasonality	**−0.487**	**−0.630 to −0.343**	**<0.001**	**−**0.183	**−**0.591 to 0.225	0.380	0.019	**−**0.005 to 0.042	0.121
Model 4: +autocorrelation	**−0.486**	**−0.633 to −0.339**	**<0.001**	**−**0.183	**−**0.602 to 0.236	0.392	0.019	**−**0.006 to 0.042	0.131

Significant associations are indicated in **bold** (p<0.05).

*Age, gender and social grade.

PoS, point of sale.

Table 4 shows the results for manual and non-manual occupations (social grade) separately. The steeper decline in smoking after the introduction of the ban was only significant in those with manual occupations (−0.62% change, 95% CI −0.93 to −0.30, p<0.001); however, the difference between manual and non-manual occupations was not significant (p for interaction=0.075). For cigarette consumption, results were similar for manual and non-manual occupations.

**Table 4:** Results of the segmented regression analysis for current smoking and cigarette consumption, stratified by social grade

	Trend pre-PoS display ban			Step level change			Change in trend		
Current smoking (log binomial)	Percentage change	95% CI	p Value	Percentage change	95% CI	p Value	Percentage change	95% CI	p Value
Non-manual occupation (A,B,C1)	**−3.72**	**−6.52 to −0.84**	**0.012**	**−**8.05	**−**15.8 to 0.40	0.061	**−**0.42	**−**0.90 to 0.06	0.084
Manual occupation (C2,D,E)	**−**0.44	**−**2.29 to 1.44	0.645	**−**1.82	**−**6.91 to 3.55	0.500	**−0.62**	**−0.93 to −0.30**	**<0.001**
Interactions with social grade			0.099			0.841			0.072
Cigarette consumption	β	95% CI	p Value	β	95% CI	p Value	β	95% CI	p Value
Non-manual occupation (A,B,C1)	**−0.390**	**−0.635 to −0.145**	**0.018**	**−**0.167	**−**0.552 to 0.886	0.649	**−**0.008	**−**0.031 to 0.047	0.689
Manual occupation (C2,D,E)	**−0.520**	**−0.701 to −0.340**	**<0.001**	**−**0.333	**−**0.838 to 0.893	0.172	0.022	**−**0.007 to 0.052	0.142
Interactions with social grade			0.595			0.415			0.295

The table presents trends previous to the introduction of the PoS display ban, the immediate step level change, and the change in the trend after the PoS ban was introduced compared with the pre-PoS display ban trend.

Significant associations are indicated in **bold** (p<0.05).

PoS, point of sale.

## Discussion

### Key findings

There was no immediate step level change in current smoking after the introduction of the PoS display ban. When taking into account sociodemographic factors, e-cigarette use, secular trend, seasonality and autocorrelation there was a significantly greater decrease in current smoking after the introduction of the tobacco PoS display ban than before the ban. We found some evidence that this change in trend was stronger among individuals with manual occupations than in those with non-manual occupations. No step level change or effects on trend were found for cigarette consumption.

### Limitations and strengths

The pre–post-PoS display ban evaluation design used in this study does not guarantee that the observed changes in trends were due to the investigated policy, since other events may have occurred and there was no control group. Changes in tobacco control in England between 2009 and 2015 include the introduction of pictorial warnings on the back of the pack in October 2009, the extension of pictorial health warnings from manufactured cigarette packs to all tobacco products in October 2010, tobacco tax increases of 2% above inflation rate in 2011, 2013 and 2014, and the ban on sale of tobacco from vending machines in October 2011. We do not expect these policies to have caused the observed change in trend because these were mostly introduced well before the PoS display ban or were reoccurring (tax increase), and will therefore be accounted for by seasonal correction.

A potential interfering factor is the tobacco tax increase of 5% above inflation rate at the end of March 2012. However, we believe that this was unlikely to have caused our results: according to the Nielsen tobacco sales data described in this study, the real price paid for a pack of 20 cigarettes increased by £0.17 when March 2012 real price was compared with April 2012, while in the other years, this increase was comparable (2011: £0.26; 2013: £0.13). Thus, the price increase was reoccurring and therefore taken into account by adjusting for seasonality. The fact that the price increase in 2012 was not higher than that in 2011 might be explained from the perspective of the consumer: smokers switch to a cheaper brand, and the perspective of the tobacco industry: absorbing taxes into tobacco sale prices (‘undershifting’^39^).

Spending on mass media campaigns was not accounted for. There was a moratorium on spending on the major channels of advertising from April 2010 to September 2011 and after the end of the moratorium, spending was markedly reduced compared to the period before April 2010.^34^ Mass media spending was stable in the years after the partial PoS display ban was introduced (Public Health England, personal communication, 13 July 2015). The temporal pattern, that took place mostly before the PoS display ban, made it very unlikely that this could account for any change pre versus post-PoS display ban.

Strengths of the data used in this study include the relatively long follow-up period after the partial PoS display ban was introduced (almost 3 years), the large and representative study sample, and the use of monthly data. Furthermore, strengths in the statistical method include the sophisticated assessment of trends before and after the introduction of the display ban as well as comparing of the levels of smoking before and after the ban, and the taking of the secular trend, seasonality and autocorrelation into account.

### Interpretation and comparison of results

We found declining overall trends in smoking prevalence and in cigarette consumption that remained consistent after controlling for sociodemographic factors. The data in the current study are recent and there is, therefore, limited comparability with previous studies, but trends in the period 2003 to 2013 are in line with trends found in previous studies of adult smokers and non-smokers in England and Britain.^40^ ^41^

The decline in smoking prevalence after the introduction of the partial PoS tobacco display ban in this study is mostly in line with results found in previous studies. Effects on smoking in the current study were less profound than that in young people in Australia.^15^ However, the study on young people in Australia did not control for the secular trend in smoking, and part of the effect observed in that study may, therefore, not be attributable to the PoS display ban.^15^ Based on the limited available evidence, a SimSmoke simulation model projected that a PoS display ban in the USA in combination with the removal of tobacco advertising at the PoS could lead to a reduction in smoking prevalence of 0.63% per year in the first 6 years.^42^ The current study suggests that the reduction in smoking over and above the secular trend was 0.46% per year. This reduction may be due to a decrease in impulse cigarette purchase in smokers.^17^ ^18^ This is likely to occur mostly in smokers who recently attempted to quit or who intend to quit, because they are more likely to make impulse tobacco purchases than smokers who did not attempt or intend to quit.^6^ ^43^ Through the reduction of impulse purchases in this group of recent ex-smokers, their chances of successful long-term quitting might have been substantially improved. It is less likely, but not ruled out, that the prevention of smoking initiation caused the steeper declining trend in smoking after the ban, as smoking uptake usually occurs during adolescence.^44^

We did not find a significant reduction in cigarette consumption after the partial PoS display ban was introduced. In theory, one cue for impulse tobacco purchase is removed by PoS display bans, which reduces impulse purchase of tobacco,^17^ ^18^ and may therefore lead to lower consumption levels. However, an Australian study^43^ found that buying cigarettes on impulse was more likely in smokers who smoked less than 10 cigarettes per day than in heavier smokers. A reduction in consumption in light smokers may have not strongly affected the overall average number of cigarettes smoked per day, since they already smoke relatively few cigarettes. We were unable to study consumption in light smokers because of the cross-sectional nature of the data and low numbers of non-daily smokers.

We did not observe an immediate step change in smoking or cigarette consumption after the introduction of the ban. This suggests that the PoS display ban did not actively encourage a large cohort of smokers to quit immediately, but instead removed a cue to purchase cigarettes and removed advertisement. Therefore, the effect of this passive measure is likely to have only taken effect once smokers tried to quit^6^ ^43^ and to have taken place over time. Moreover, the cue for tobacco purchase was not fully removed, because the ban was partial. This is particularly true if the cupboard where the tobacco is held is labelled prominently with the word ‘tobacco’.^45^ The effects may, therefore, be expected to be stronger for a comprehensive ban that does not allow for large textual cues.

The partial PoS display ban's impact on a change in trend was significant in individuals with manual occupations but not those with non-manual occupations. We were unable to form an a priori hypothesis on the equity impact of the partial PoS display ban due to the lack of evidence. However, since most national tobacco control policies have been found to be equity neutral or negative,^20^ the result of a suggested equity positive effect was unexpected. Since the investigated PoS ban was partial, only implemented in larger shops, a difference in exposure to the ban by social grade may have played a role in differential effects. There have been very few studies assessing determinants of tobacco purchasing behaviour,^43^ and a potential difference in exposure by social grade needs further exploration.

### Conclusions

The implementation of a partial tobacco PoS display ban was not associated with an immediate step change in smoking, but was associated with a stronger decline in smoking after the introduction of the display ban, over and above the secular trend and seasonal factors. We found some evidence that the observed effect of the partial tobacco PoS display ban was stronger in individuals with manual occupations than in those with non-manual occupations. This study did not provide evidence for a decrease in cigarette consumption among smokers as a result of the PoS display ban.

**What this paper adds**

- The partial tobacco point of sale display ban introduced in England was associated with a decline in smoking prevalence over and above the secular trend and any seasonal factors.
- The partial tobacco point of sale display ban may have had a larger effect on smoking behaviour of lower socioeconomic groups than higher socioeconomic groups.
- This study did not find evidence of a decrease in cigarette consumption among smokers as a result of the point of sale display ban.
